# Supplementary material for: A new pipeline SPICE identifies novel JUN-IKZF1 composite elements
Source: bioRxiv. 2024 Dec 12:2023.05.31.543110. Preprint. [Version 2] doi: 10.1101/2023.05.31.543110 (PMC11703198; doi:10.1101/2023.05.31.543110)
Supplement: Supplement 2 [file media-2.pdf]

Table 1

STATs (Conditions)	Identified canonical GAS motif	GAS%	Tetramer likelihood	Optimal Spacing (bp)
STAT1 (MΦ, +IFN-γ)	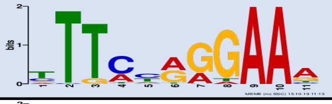	87.4%	****	10
STAT2 (Th1, +IFN-γ)	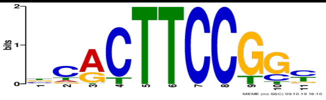	38.2%	NA	NA
STAT3 (CD8T, +IL-21)	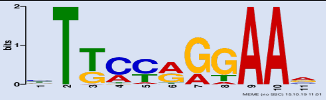	87.0%	***	11?
STAT4 (Th1)	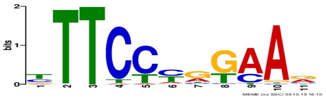	95.4%	****	11-12
STAT5A (Total T, +IL-2)	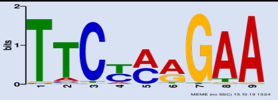	99.8%	****	6-7, 11-12
STAT5B (Total T, +IL-2)	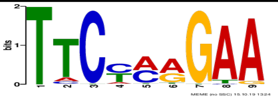	100%	****	6-7,11-12
