# Supplementary figures and images for: A new pipeline SPICE identifies novel JUN-IKZF1 composite elements
Source: bioRxiv. 2024 Dec 12:2023.05.31.543110. Preprint. [Version 2] doi: 10.1101/2023.05.31.543110 (PMC11703198; doi:10.1101/2023.05.31.543110)

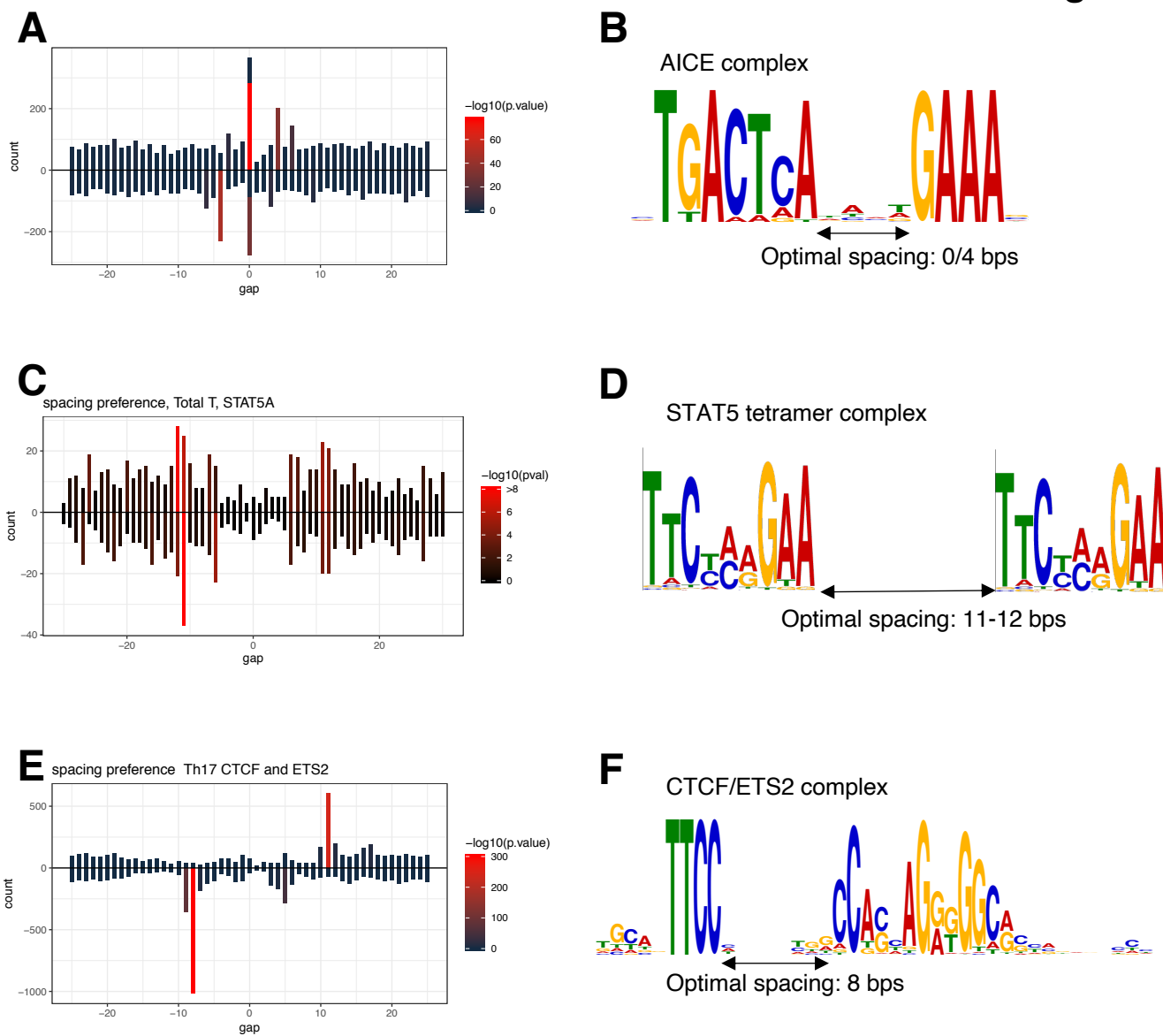

Figure S2

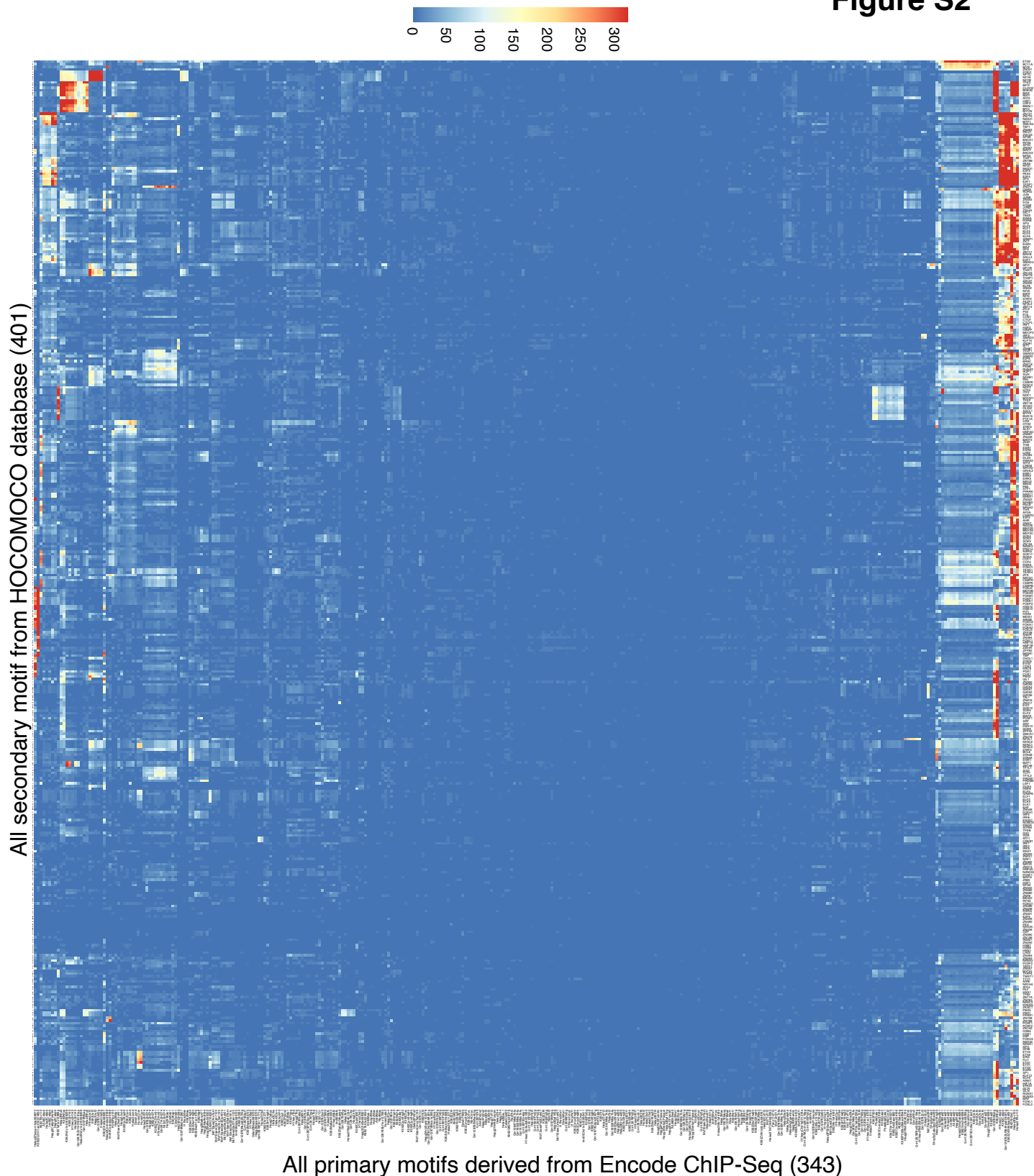



Figure S4

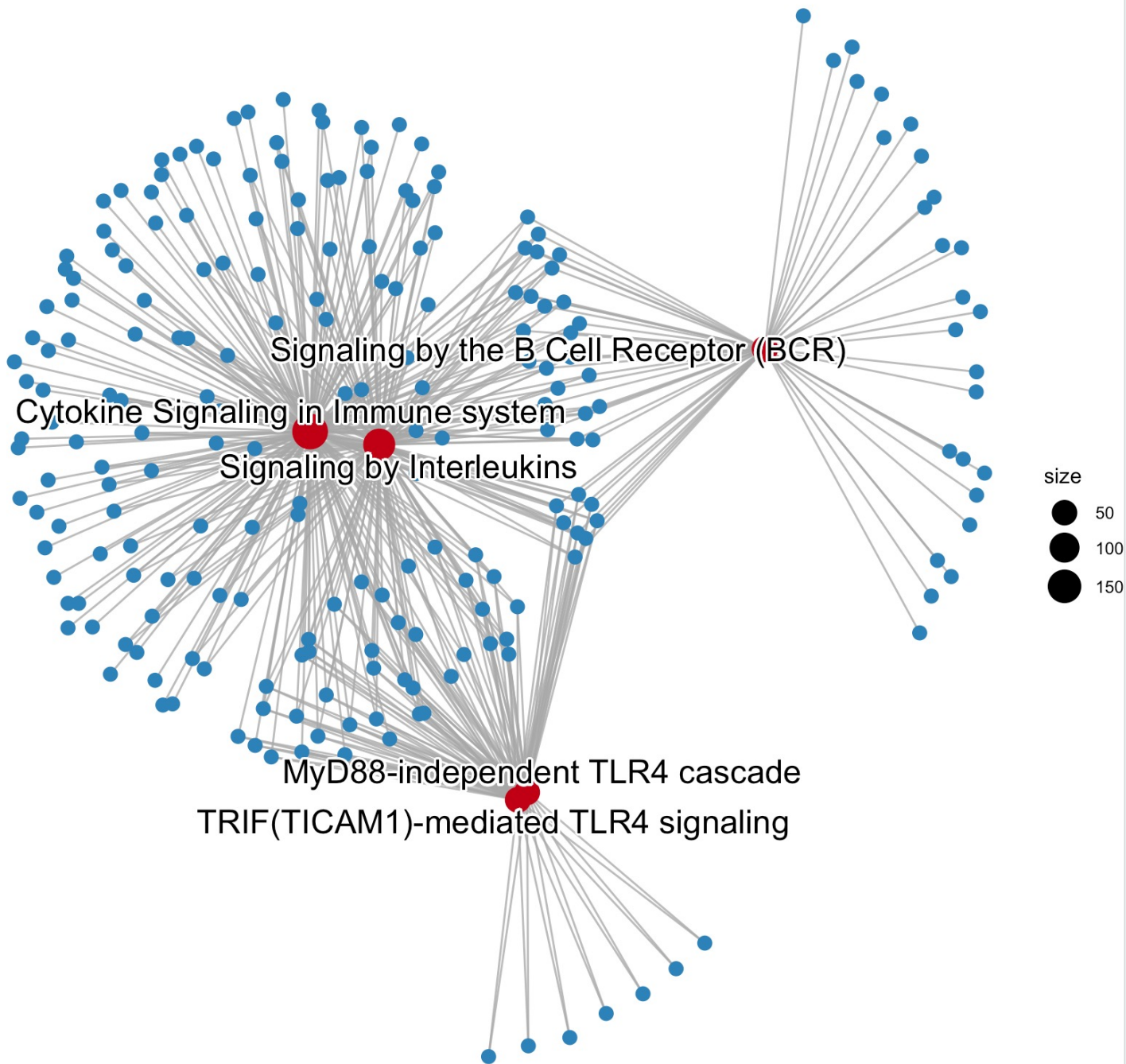

Supplement: 3 — Figure S1. SPICE successfully predicts AICE, STAT5 tetramers, and CTCF/ETS complexes. SPICE successfully predicted previously identified composite elements, including AICE (AP-1/IRF4 composite elements), based on BATF, JUN and IRF4 ChIP-Seq data in TCR pre-activated mouse T cells (A, B), STAT5 tetramer complex formation based on STAT5 ChIP-Seq in TCR pre-activated and IL-2-treated T cells (C, D), and CTCF/ETS composite elements based on available ETS2 ChIP-Seq data (E, F). Figure S2. SPICE predicts transcription factor composite elements using the Encode ChIP-Seq data. Heat map of the motif interaction matrix of ChIP-Seq libraries from Transcription Factor Binding Sites (TFBSs) from the ENCODE project. The x-axis represents the primary motifs derived from 343 Encode ChIP-Seq libraries, and the y-axis indicates the 401 motifs from HOCOMOCO database (these are also in Table 2). The color scale represents the −log10 transformed E-value, which is the lowest p-value of any spacing of the secondary motif times the number of secondary motifs. Figure S3. JUN-IKZF composite elements in GM12878 cells. (A) Heat map of genome-wide ChIP-Seq binding intensities in a ± 3 kb genomic region centered on summits of combined IKZF1 and JUN peaks in GM12878 cells from the ENCODE project; two biological replicate experiments are shown in the heat map. (B) Co-localization of IKZF1, JUN, and BATF peaks at the IL10 and TNFRSF8 genomic loci. A conserved cis-regulatory element in the human IL10 and mouse Il10 loci, CNS9, is highlighted in the red box. (C) Sequence alignment reveals CNS9 region is highly conserved in human and mouse. Figure S4. Gene-concept network depicts the linkages of genes and biological concepts of the top 5 Reactome Pathways that were enriched in IKZF1-JUN shared peaks. The highlighted red dots represent the top 5 enriched pathways and the dot size indicates the number of genes involved in each pathway. [file NIHPP-2023.05.31.543110V2-supplement-1.pdf]
